# Crystal structure of human NLRP12 PYD domain and implication in homotypic interaction

**DOI:** 10.1371/journal.pone.0190547

**Published:** 2018-01-02

**Authors:** Tengchuan Jin, Mo Huang, Jiansheng Jiang, Patrick Smith, Tsan Sam Xiao

**Affiliations:** 1 Laboratory of structural immunology, CAS Key Laboratory of innate immunity and chronic diseases, CAS Center for Excellence in Molecular Cell Science, School of Life Sciences and Medical Center, University of Science and Technology of China, Hefei, Anhui, PRC; 2 Structural Immunobiology Unit, Laboratory of Immunology, National Institute of Allergy and Infectious Diseases, National Institutes of Health, Bethesda, Maryland, United States of America; 3 Department of Pathology, Case Western Reserve University, Cleveland, Ohio, United States of America; Russian Academy of Medical Sciences, RUSSIAN FEDERATION

## Abstract

NLRP12 is a NOD-like receptor that plays multiple roles in both inflammation and tumorigenesis. Despite the importance, little is known about its mechanism of action at the molecular level. Here, we report the crystal structure of NLRP12 PYD domain at 1.70 Å fused with an maltose-binding protein (MBP) tag. Interestingly, the PYD domain forms a dimeric configuration through a disulfide bond in the crystal. The possible biological significance is discussed in the context of ROS induced NF-κB activation.

## Introduction

NOD-like receptors (NLRs) belong to a large multi-domain protein family and have prominent biological functions. Many NLRs have been characterized as pattern recognition receptors that sense microbial products in the cytoplasm of cells. Among them, NLRP1, NLRP3, NLRC4, NLRP6, NLRP10, NLRP12, NOD1 and NOD2 are best-studied members, and many of them function through a caspase-1 activating protein complex, called the inflammasomes [1]. The NLR family proteins play critical roles in many inflammatory and autoimmune diseases [2].

NLRP12 is a NLR family member that received much attention recently. NLRP12 is mainly expressed by the immune cells, and its expression is down-regulated in response to pathogen products and inflammatory cytokines. It has been predicted to play a role as a negative regulator of the inflammatory response [3]. Mutations in NLRP12 have been described in patients affected with peculiar autoinflammatory symptoms [4, 5]. Jera et al reported that the NLRP12-associated autoinflammatory disorders in the NLRP12 mutant-carrying patients were mediated by constitutive secretion of interleukin-1β (IL-1β) [6]. The NLRP12 inflammasome was reported to play an important role in the recognition of *Yersinia pestis*, the causative agent of plague, through controlling interleukin-18 (IL-18) and IL-1β production [7]. In addition to the previously reported function in inflammation, recent studies showed that NLRP12 was involved in tumorigenesis. Zaki et al reported that NLRP12 could attenuate colon inflammation and tumorigenesis by maintaining intestinal homeostasis through dampening nuclear factor-kappa B (NF-κB) and extracellular signal-regulated kinase (ERK) activation in macrophages [8, 9]. A recent study by Chen et al further found that NLRP12 attenuates colon inflammation by maintaining colonic microbial diversity and promoting protective commensal bacterial growth [10].

The significance of NLRP12 in both chronic inflammation and cancer warrant extensive structure-function characterization of this protein and its mutants. Most of the NLRPs proteins contain an N-terminal Pyrin domain (PYD), a central nucleotide oligomerization domain (NOD) and a C-terminal Leucine-rich repeats (LRR). The PYD domain belongs to a large protein superfamily called death domain superfamily together with the Death Domain (DD), the Death Effector Domain (DED) and the Caspase Recruitment Domain (CARD). Death domain fold containing proteins play pivotal roles in apoptosis, necrosis and inflammation. Many of these members function through homotypic interactions that mediate the formation of oligomeric structures [11, 12].

Despite the report of an NMR structure of the NLRP12 PYD [13], its homotypic interaction interface has not been mapped. The interaction interface between the NLRP12 PYD and the adaptor protein ASC is not characterized. Little is known about how NLRP12 functions through interaction with itself or other proteins. In this study, we determined a 1.70 Å crystal structure of the human NLRP12 PYD fused with an N-terminal maltose binding protein (MBP). We observed a homotypic PYD: PYD dimer interaction mediated by an intermolecular disulfide bond in the crystal lattice. The possible biological function of this dimer is further discussed.

## Methods

### Protein expression and purification

Coding sequence for residues L10-S105 of the human NLRP12 (NCBI accession # NP_653288) was cloned into a pET30a expression vector (Novagen, Madison, WI) with a non-cleavable N-terminal MBP tag. The MBP tag contains surface entropy reducing mutations D82A/K83A/E172A/N173A/K239A to enhance crystallization probability [14, 15]. A continuous rigid helical linker (AARAFAAA) was added between the last helix of MBP at N368 and the first helix of PYD at L10. Transformed BL21 (DE3) Codon Plus RIPL cells (Stratagene, Santa Clara, CA) were grown at 37°C until OD600 reached 1.2. Cells were then induced with 0.2 mM IPTG at 18°C for 4 hours, harvested and resuspended in buffer A (20 mM Tris-HCl, pH 8.0, 100 mM NaCl) plus 5 mM imidazole and supplemented with DNase (Biomatik, Wilmington, DE) and protease inhibitors (Roche Applied Science, Indianapolis, IN). Cells were lysed by sonication, and soluble protein was purified from cell lysate by Hisprep IMAC column (GE Healthcare Bio-Sciences, Piscataway, NJ). IMAC column was eluted with a 75 ml linear gradient of elution buffer (500 mM NaCl, 200 mM Imidazole, 20 mM Tris-HCl pH 8.0). 5 mM DTT and 2 mM EDTA was added to the IMAC eluted MBP-PYD protein, and was further purified by a XK26/60 Superdex 200 (GE Healthcare Bio-Sciences, Piscataway, NJ) gel filtration column in buffer B (100 mM NaCl, 2mM DTT, 20 mM Tris-HCl pH 8.0) supplemented with 5 mM maltose (Research Products International Corp, Mount Prospect, IL). The MBP-tagged PYD protein elutes as a monomer.

### Crystallization

Purified MBP-PYD protein in buffer B was concentrated by Amicon centrifugal concentrators (Millipore, Billerica, MA) to 20 mg/ml before setting up hanging drop vapor diffusion method for crystallization. Multiple commercially available crystal screens were tested using the Mosquito crystallization robot (TTP Labtech, United Kingdom). Single crystals grew after one week with a well solution containing 3.5 M sodium formate, 0.1 M sodium acetate, pH 4.6. 20% glucose (w/v) was added to the reservoir solutions as the cryoprotectant to flash cool the crystals in liquid nitrogen for X-ray diffraction data collection.

### X-ray diffraction, structure determination and refinement

X-ray diffraction data were collected at GM/CA beamline at the Advanced Photon Source, Argonne National Laboratory (ANL). Data were processed with HKL2000 program suite [16] and XDS [17]. The best crystal diffracted to 1.70 Å. The space group is P2_1_2_1_2 with unit cell parameters a = 103.62 Å, b = 186.74 Å, c = 52.72 Å (also see Table 1). The Matthews coefficient is 2.41 Å^3^ Da^-1^, which suggests two molecules of fusion protein in each asymmetric unit with an estimated solvent content of 49.0%. The structure was determined by molecular replacement with Phaser [18] in the CCP4 program suite [19]. The MBP structure from PDB 3VD8 [20] and the NMR structure of the NLRP12 PYD (PDB: 2L6A) [13] was used as search models in a two-step search. The final model was completed by alternative manual model building in Coot [21] and Phenix.refine refinement in Phenix GUI [22]. The crystal structures were validated by the Molprobity server [23] and RCSB ADIT validation server [24]. Most of the values in Table 1 were generated by the Table 1 utility in the Phenix package. Electrostatics surfaces were calculated with program APBS[25] with PDB2PQR [26] using the AMBER force yield and displayed with Pymol (Delano Scientific LLC, San Carlos, CA). Protein sequence alignment was prepared with ClustalW [27] with manual adjustments of gaps.

**Table 1:** X-ray data collection and refinement table.

**Data Collection**	MBP-NLRP12 PYD
Spacegroup	P2_1_2_1_2
Unit cell (a, b, c) (Å)	103.62, 186.74, 52.72
(α, β, γ) (°)	90, 90, 90
Wavelength (Å)	0.97
Wilson B-factor (Å^2^)	19.4
Anisotropy	0.242
Resolution (last shell) (Å)	50–1.70 (1.80–1.70) *
No of reflections (total/unique)	670878/110662
Completeness (%)	97.5 (85.3) *
Average multiplicity	6.1 (3.1) *
I/σ(I)	22.4 (3.7) *
Rmerge (%)^¶^	5.1 (28.7) *
**Refinement**	
Resolution (Å)	50–1.70
No. of protein atoms/ average *B*-factor (Å^2^)	7757/ 25.17
No. of hetero atoms/ *B*-factor (Å^2^)	564/ 34.93
Rmsd bond lengths (Å)	0.007
Rmsd bond angles (°)	1.00
Rwork^†^	0.160
Rfree^‡^	0.201
Ramachandran plot favored/disallowed (%)**	98.8 /0
PDB code	4XHS

^¶^ R_merge_ = Σ_h_ Σ_i_ |I_*i*_(*h*) -<I(*h*)> | / Σ_h_Σ_i_ I_i_(*h*), where I_i_(*h*) and <I(*h*)> are the ith and mean measurement of the intensity of reflection *h*.

^†^ R_work_ = Σ_h_||*F*_obs_ (*h*)|-|*F*_calc_ (*h*)|| / Σ_h_|*F*_obs_ (*h*)|, where *F*_obs_ (*h*) and *F*_calc_ (*h*) are the observed and calculated structure factors, respectively. No I/σ cutoff was applied.

^‡^R_free_ is the R value obtained for a test set of reflections consisting of a randomly selected 5% subset of the data set excluded from refinement.

**Values from Molprobity server (http://molprobity.biochem.duke.edu/).

### Protein Data Bank accession codes

The coordinates and structural factors of MBP-PYD fusion protein have been deposited in the Protein Data Bank with accession code 4XHS.

## Results

### Quality of structure

Our initial effort to crystallize the human NLRP12-PYD domain did not identify any promising conditions of crystallization that could yield crystals suitable for X-ray data collection. As a result, we tried an alternative strategy of using the bacterial MBP as a crystallization tag to facilitate the crystallization of the PYD domain. This approach of using MBP as a crystallization chaperone has led to structural determination of many proteins from the death fold superfamily and other protein families [15, 20, 28–30].

**Fig 1:**
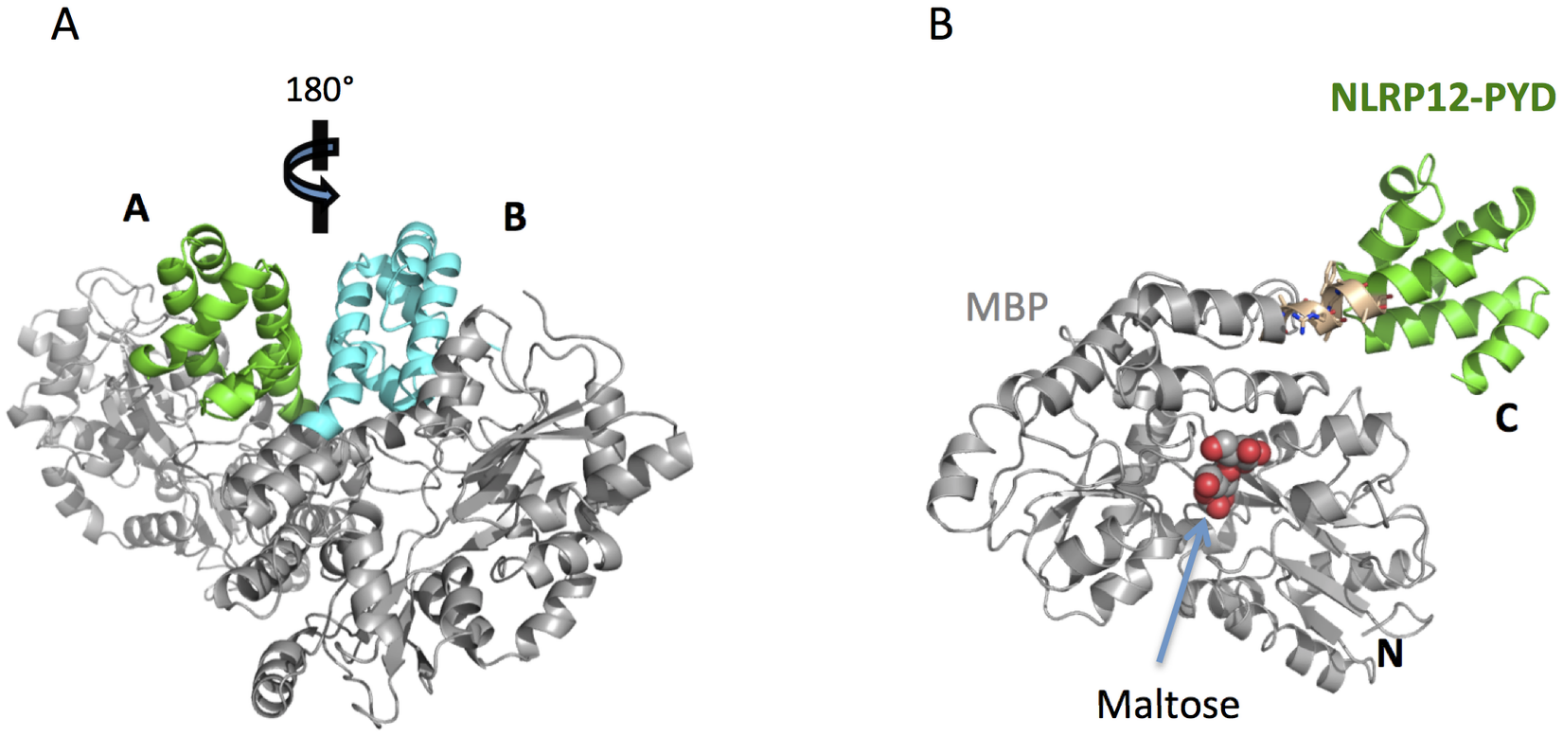
Crystal structure of MBP-PYD. (A) Cartoon representation of a crystallographic asymmetric unit containing two copies of the MBP-PYD fusion protein. The MBPs are colored in grey, and the PYDs in chains A and B, are colored in green and cyan, respectively. (B) MBP-PYD structure in chain A. The amino terminus is labeled by “N” and the Carboxyl terminus is labeled by “C”. The linker region is colored in wheat and shown in sticks. The maltose molecule bound to MBP is shown in spheres.

In this study, we fused the PYD domain of the human NLRP12 to the C-terminus of the MBP mutant with a designed helical linker sequence (see Method). The MBP-tagged PYD protein elutes as a monomer in gel filtration. The fusion protein was crystallized in the P2_1_2_1_2 space group (see Table 1). There are two copies of the fusion proteins in the crystallographic asymmetric unit (Fig 1A). At a resolution of 1.70 Å, NCS was not applied in the final stages of refinement using Phenix, because it did not further improve the fit between the model and the electron density (data not shown). In the final model, the two protein chains include 452 and 454 residues respectively. Residue L377 in the fusion protein corresponds to L10 of the NLRP12 PYD (NCBI accession # NP_653288). Some residues in the loop region between helix 2 and helix 3 are not included in the final model because of diffusive electron density in these regions, which suggests this region may adopt multiple conformations as revealed by the published NMR structure [13]. Multiple sidechain rotamers were observed in some residues and alternative conformations were included in the final model. Furthermore, there are two maltose (MAL) molecules, four formate (FMT) molecules, five sodium (Na) ions, and 1081 water (HOH) molecules in the final model. All of the data to 1.70 Å resolution were used in the final refinement, with Rwork = 0.1604 and Rfree = 0.2009. Molprobity validation shows 98.81% of all protein residues are in favorite region and there are no outliers in the Ramachandran plot (Table 1). There are two sidechain rotamer outliers, which accounts for 0.5% of total residues. The average B-factor is 25.5 Å^2^ for all 8322 atoms included in the final model.

### Overall structure of PYD

As expected, the linker region between the N-terminal MBP and C-terminal PYD adopts an α-helix conformation. The linker helix is slightly bent, which allows the PYD domain to be located away from the MBP such that there is no significant contact between them (Fig 1B). The bending is likely caused by the interaction of the two PYDs in the dimer (discussed below).

**Fig 2:**
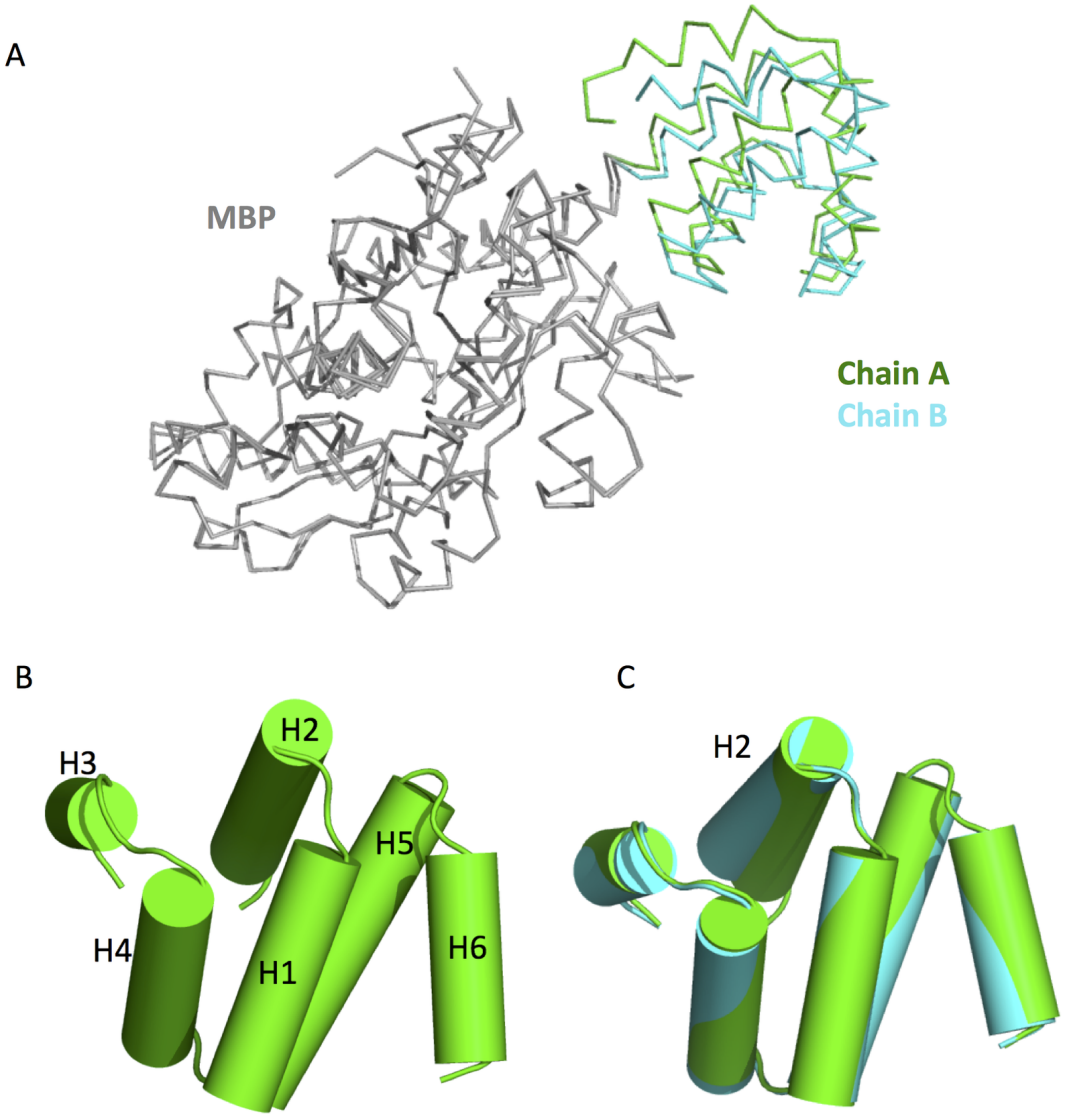
Structural comparison of chain A and chain B. (A) Superposition of chain A and chain B. The MBP regions of the two chains were aligned. (B) Structure of the NLRP12 PYD domain. A. The 6 helices are labeled as H1-H6. (C) Superposition of PYD domain in chain A and chain B.

Chain A and chain B is related by a non-crystallographic symmetry (Fig 1A). Superposition of all residues in chains A and B shows an rmsd of ~ 0.23 Å, which results from conformational difference between the MBP and PYD in each fusion protein. Fig 2A shows that upon superposition of the MBPs from chain A and chain B, the C-terminal PYDs adopt slightly different conformations.

The NLRP12-PYD has a globular structure of a six-helix bundle similar to other members of the death domain superfamily (Fig 1A). The NLRP12 PYD has a short H3-helix (Fig 2B), which is a characteristic feature of the PYD subfamily [31]. Superposition of the PYDs from both chains shows an rmsd of 0.30 Å (Fig 2C). Most of the 5 helices could be perfectly superimposed with the exception of the H2 helix. The loop region connecting H2 and H3 (L23), could not be built in the electron density map, suggesting that this region is highly flexible. It was also in agreement with the recent NMR study, which showed that the L23 loop is the most variable region in the structure [13].

### Structural comparison with other PYDs

This 1.70 Å resolution structure solved by X-ray crystallography is very similar to the structure solved by NMR (PDB 2L6A). The NMR structure can be superimposed over the PYD region of chain A and chain B with rmsd of 1.15 Å to 1.28 Å.

**Fig 3:**
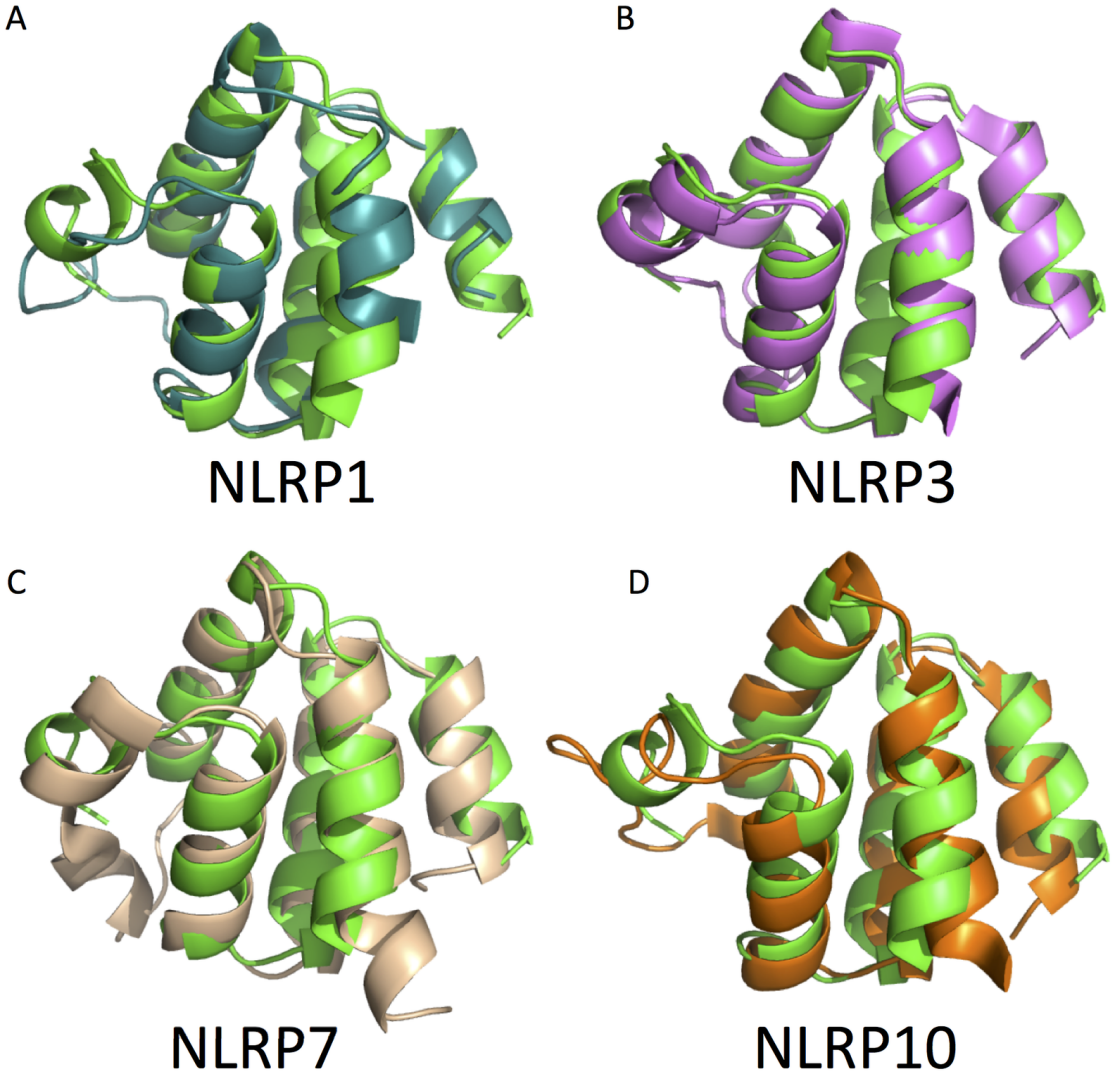
Structural comparison with other NLRP PYD domains. The PYD structures of NLRP1 (PDB: 1PN5, color: teal), NLRP3 (PDB: 3QF2, color: violet), NLRP7 (PDB: 2KM6, color: wheat) and NLRP10 (PDB: 2DO9, color: orange) were superimposed onto our NLRP12 PYD chain A.

The PYD structures of four other NLRP proteins have been solved previously including that of NLRP1 [32], NLRP3 [33], NLRP7 [34] and NLRP10 [35]. After structural comparison, we found that the NLRP3 PYD is the most similar structure to our NLRP12 crystal structure, with an rmsd of 0.78 Å (see Fig 3).

### Identification of a disulfide-mediated homotypic interaction interfaces

**Fig 4:**
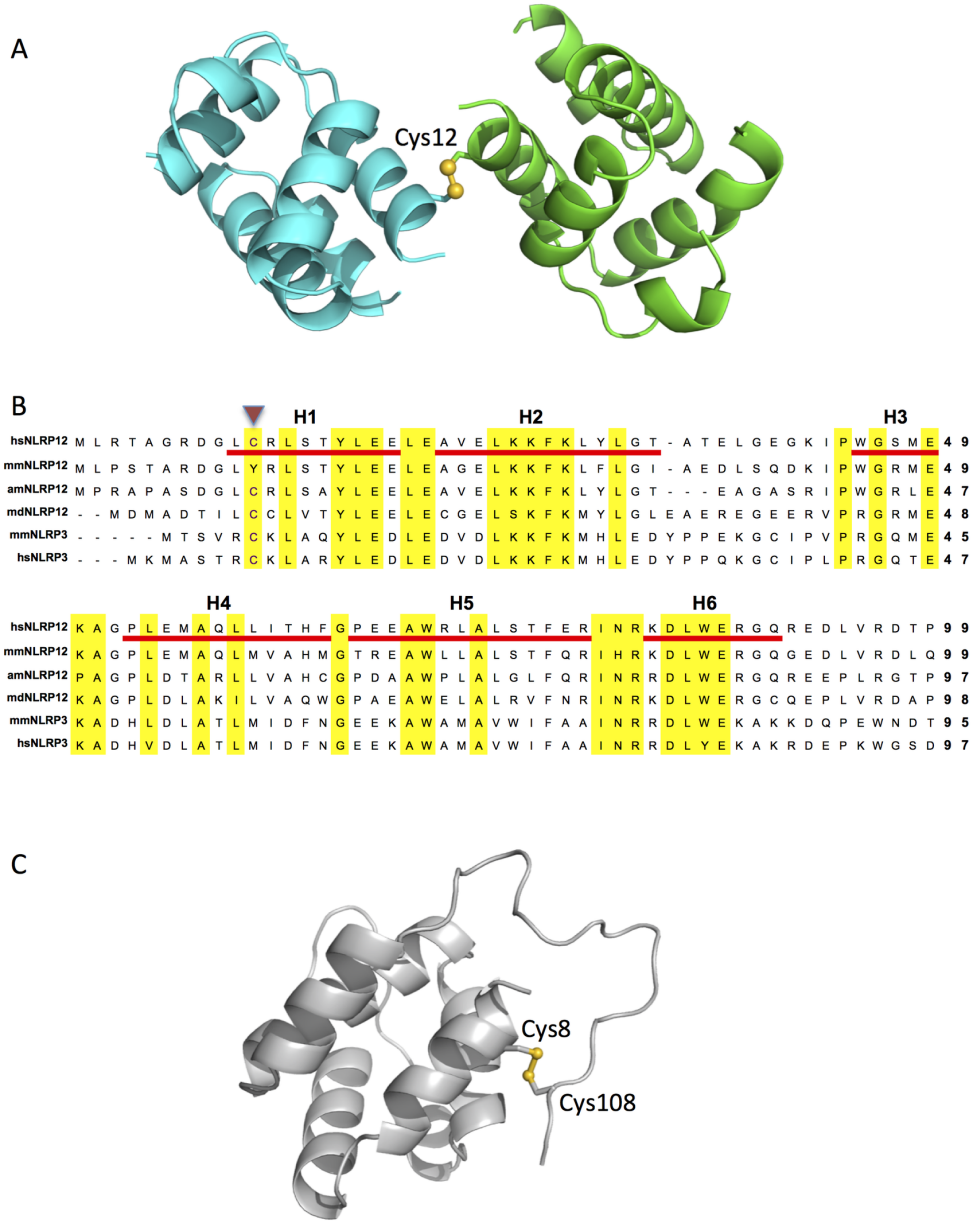
Disulfide bond-mediated dimerization. (A) The disulfide bond is formed between the Cys11 residues from chain A and chain B. It is colored in gold. (B) Sequence alignment of the PYD region of selected NLRP12 and NLRP3 proteins. The helices were labeled as H1- H6. The conserved region are colored with yellow background. The conserved cysteine residues are indicated with a purple triangle. (C) Intra-molecular disulfide bond of human NLRP3 PYD. An intra-molecular disulfide bond between Cys8 and Cys108 was observed in the reported crystal structure of the human NLRP3 PYD (PDB: 3QF2).

Interestingly, in the asymmetric unit, we identified a ‘dimer’ of fusion proteins. The dimer was mediated by an intermolecular disulfide bond formed between the Cys11 residues of the PYD domains (Fig 4A). There are no other residues involved in the dimer interface. Considering the fact the PYD domain is a monomer after purified in a reducing environment (data not shown), we think this disulfide bond formation is the major driving force for this dimer configuration.

## Discussion

The current study identifies an exposed cysteine residue Cys11 at the N-terminal PYD domain of the human NLRP12 protein that can mediate disulfide bond formation. Although this intriguing finding is preliminary, we speculate that this PYD domain can interacte with other PYD domains or other proteins/domains through this exposed cysteine. Indeed, this Cys11 of human NLRP12 is conserved in NLRP12 and NLRP3 proteins (Fig 4B). Interestingly, in the recently reported crystal structure of the human NLRP3 PYD domain, this equivalent free cystein in H1 formed a disulfide bond with a cystein residue in the loop region following H6 (Fig 4C) [33]. In addition, disulfide bond mediated dimerization was observed in the CARMA1 CARD, another subfamily of the death fold superfamily [36]. These examples suggest that such free cystein is highly reactive, and can potentially mediate self-association or interaction with another protein to modulate biological functions [37, 38].

Even though the mechanism for such disulfide formation in vivo is not well defined, it was proposed that reactive oxygen species (ROS) is a cancer inducing agent, although its mechanism is less clear [39]. There are several previous studies on the crosstalk between the ROS and the NF-κB pathways [40, 41]. Combining with the recent discovery of the inhibitory role of NLRP12 on inflammation through a noncanonical NF-κB signaling pathways, we propose that it is plaussible that excessive tissue ROS level can induce the formation of the intermolecular disulfide bond in NLRP12 that resembles the crystallographic dimer reported here. The dimeric NLRP12 may modulate the noncanonical NF-κB signaling pathway and contribute to the induction of cancer. Obviously ROS may impact other facets of cellular physiology to induce carcinogenesis, therefore our hypothesis based on the observation of a crystallographic dimer must await future studies of NLRP12 under physiological conditions. For example, a cysteine to serine mutation of the NLRP12 PYD can be knocked-in to replace the wild type protein, and the role of this mutant NLRP12 in colon inflammation and cancer can be evaluated in animal models reported by Zaki et al [8, 9] and Chen et al [10].
